# SplitFusion enables ultrasensitive gene fusion detection and reveals fusion variant-associated tumor heterogeneity

**DOI:** 10.1016/j.patter.2025.101174

**Published:** 2025-02-14

**Authors:** Weiwei Bian, Baifeng Zhang, Zhengbo Song, Binyamin A. Knisbacher, Yee Man Chan, Chloe Bao, Chunwei Xu, Wenxian Wang, Athena Hoi Yee Chu, Chenyu Lu, Hongxian Wang, Siyu Bao, Zhenyu Gong, Hoi Yee Keung, Zi-Ying Maggie Chow, Yiping Zhang, Wah Cheuk, Gad Getz, Valentina Nardi, Mengsu Yang, William Chi Shing Cho, Jian Wang, Juxiang Chen, Zongli Zheng

**Affiliations:** 1 Ming Wai Lau Centre for Reparative Medicine, Karolinska Institutet, Hong Kong SAR, China; 2 Department of Medical Epidemiology and Biostatistics, Karolinska Institutet, 171 77 Stockholm, Sweden; 3 Department of Clinical Trial, Zhejiang Cancer Hospital, Hangzhou, Zhejiang 310022, China; 4 Hangzhou Institute of Medicine (HIM), Chinese Academy of Sciences, Hangzhou, Zhejiang 310018, China; 5 Broad Institute of MIT and Harvard, Cambridge, MA, USA; 6 The Mina and Everard Goodman Faculty of Life Sciences, Bar-Ilan University, Ramat Gan, Israel; 7 Department of Biomedical Sciences and Tung Biomedical Sciences Centre, City University of Hong Kong, Hong Kong SAR, China; 8 Department of Pathology, Fujian Cancer Hospital, Fuzhou 350014, China; 9 Department of Precision Diagnostic and Therapeutic Technology, City University of Hong Kong Shenzhen Research Institute, Shenzhen, China; 10 Department of Neurosurgery, Shanghai Changhai Hospital, Shanghai 200003, China; 11 Department of Pathology, Queen Elizabeth Hospital, Hong Kong SAR, China; 12 Department of Pathology, Massachusetts General Hospital, Boston, MA, USA; 13 Center for Cancer Research, Massachusetts General Hospital, Boston, MA, USA; 14 Harvard Medical School, Boston, MA, USA; 15 Department of Clinical Oncology, Queen Elizabeth Hospital, Hong Kong, China; 16 International Peace Maternity and Child Health Hospital, School of Medicine, Shanghai Jiaotong University, Shanghai 200030, China

**Keywords:** fusion detection tool, RNA-seq, FFPE samples, tumor heterogeneity, TCGA, ALK variant, ARv7, DUX4, ROS1, AMP

## Abstract

Gene fusions are common cancer drivers and therapeutic targets, but clinical-grade open-source bioinformatic tools are lacking. Here, we introduce a fusion detection method named SplitFusion, which is fast by leveraging Burrows-Wheeler Aligner-maximal exact match (BWA-MEM) split alignments, can detect cryptic splice-site fusions (e.g., *EML4::ALK* v3b and *ARv7*), call fusions involving highly repetitive gene partners (e.g., *CIC::DUX4*), and infer frame-ness and exon-boundary alignments for functional prediction and minimizing false positives. Using 1,848 datasets of various sizes, SplitFusion demonstrated superior sensitivity and specificity compared to three other tools. In 1,076 formalin-fixed paraffin-embedded lung cancer samples, SplitFusion identified novel fusions and revealed that *EML4::ALK* variant 3 was associated with multiple fusion variants coexisting in the same tumor. Additionally, SplitFusion can call targeted splicing variants. Using data from 515 The Cancer Genome Atlas (TCGA) samples, SplitFusion showed the highest sensitivity and uncovered two cases of *SLC34A2::ROS1* that were missed in previous studies. These capabilities make SplitFusion highly suitable for clinical applications and the study of fusion-defined tumor heterogeneity.

## Highlights

• SplitFusion is an ultrasensitive, fast, and clinical-grade gene fusion detection tool
• SplitFusion supports both anchored and regular RNA-seq data
• SplitFusion can detect fusions involving highly repetitive regions
• SplitFusion reveals fusion variant-associated fusion heterogeneity

## The bigger picture

Chromosomal rearrangements are alterations of native chromosomal structures causing gene fusions, which can cause cancers. Therefore, these rearrangements are considered therapeutic targets. Most tools to detect gene fusions are designed for research and may not perform well in clinical settings. Clinical detection often relies on low-quality mRNA samples extracted from formalin-fixed paraffin-embedded (FFPE) tissues. While the focus in the clinical setting is often on detecting actionable fusion targets, increasing adoption of next-generation sequencing has led to identification of many so-called “passenger fusions,” which refers to gene rearrangements involving genes that are in proximity within the nucleus but are not functional. SplitFusion addresses these challenges and demonstrates superior sensitivity and specificity in rigorous tests with new and previously published clinical data.

## Teaser

The authors present SplitFusion, an ultrasensitive, fast, clinical-grade gene fusion detection tool. It supports both AMP-seq and RNA-seq data, demonstrating superior sensitivity and specificity. Additionally, SplitFusion offers desired clinical reporting features and reveals fusion variant-associated heterogeneity.

## Introduction

Chromosomal rearrangements or gene fusions that produce novel or overexpressed oncoproteins have been identified across different human cancers.^1^ The essential cure for leukemia harboring *BCR::ABL*^2^ and *PML::RARA*^3^ gene fusions represented an early success in the era of precision medicine. Beyond blood cancers, recurrent gene fusions have transformed the management of solid tumors, including *ALK*^4^ and *ROS1*^5^ in lung cancer, *TMPRSS2* in about 50% of prostate cancer,^6^ *DNAJB1*::*PRKACA* in 100% of fibrolamellar hepatocellular carcinoma,^7^ *FGFR*s in diverse cancers,^8^ and *NTRK* fusions, which have led to FDA-approved drugs regardless of tissue type.^9^

Clinical diagnosis of gene fusions is challenging both experimentally and computationally. The Cancer Genome Atlas (TCGA) study estimated the overall prevalence of gene fusions at about 16% overall,^10^ with 3% involving promising therapeutic targets (ranging from 0 to 12.9% across different tissue types).^11^ RNA sequencing (RNA-seq) analyses are effective in identifying novel gene fusions but often have low specificity, leading to many false positives. This is problematic for clinical diagnosis, as significant effort is required to filter out clinically insignificant gene fusions, with 37% of the identified 25,664 fusions in one study not being validated in genomic data.^10^ In addition, with the increasing use of next-generation sequencing in clinics, there has been a rise in the detection of “fusion passengers” (often “futile” rearrangements involving genes that are in close proximity within the nucleus but are not functional).^12^ Furthermore, alternative RNA splicing and exon deletion caused by alterations at the genomic level, which might be therapeutically important, such as *EGFR vIII*,^13^ are not detectable by most fusion detection tools. Various tools have been developed to detect gene fusions for next-generation sequencing (NGS) data, including commercial software and open-source tools like EricScript,^14^ Lumpy,^15^ and STAR-Fusion,^16^ with the latter being a top performer among 23 different tools.^16^ Another tool based on STAR alignment, Arriba,^17^ is not able to detect fusions involving repeats, such as *CIC-DUX4*, which is clinically relevant in sarcoma. Most previous tools are designed for analyzing high-quality RNA-seq data.^16^ However, none, to our knowledge, are specifically oriented for clinical diagnosis using formalin-fixed paraffin-embedded (FFPE) samples, which are often highly degraded. Additionally, they do not integrate all the desirable features, including (1) inferring fusion frame status (in frame or out of frame), (2) judging exon-boundary alignment (when one sequence has multiple split or “disconnected” alignments at exon-intron boundaries according to reference databases [e.g., RefSeq] for filtering functionally insignificant or clinically irrelevant fusions, (3) reporting specific or targeted alternative splicing and exon deletion events, and (4) customizing fusion calling for intrinsically challenging cases, such as those involving highly repetitive genes of clinical relevance.

We previously developed an NGS target enrichment method called Anchored Multiplex PCR (AMP), which enables scalable and efficient detection of gene fusions without prior knowledge of the fusion partners.^18^ For broad clinical and research applications, a high-performing fusion detection bioinformatic method is still required. To address this, we introduce an algorithm named SplitFusion (https://github.com/Zheng-NGS-Lab/SplitFusion), an algorithm designed for fusion detection with desired clinical reporting features. SplitFusion is also a powerful research tool for studying fusion variants and alternative splicing across different cancers and subclones within the same tumor. SplitFusion is fast by leveraging the chimeric alignments (split reads) detected by Burrows-Wheeler Aligner-maximal exact match (BWA-MEM)^19^ for RNA-seq data and demonstrates higher sensitivity and specificity than current well-established tools. Furthermore, SplitFusion features highly desirable abilities for clinical reporting. In 1,076 lung cancer and 35 glioblastoma FFPE samples, SplitFusion not only detected known common and rare fusions but also novel ones as well as intragenic exon deletions. Using standard RNA-seq from 515 TCGA lung adenocarcinoma datasets, SplitFusion showed higher sensitivity in fusion detection than three previous studies of the same dataset. Last, SplitFusion reveals a fusion junction hot zone in *EML4::ALK* variant 3 (v3) that may be associated with high intratumor fusion variant heterogeneity, as indicated by multiple fusion transcript junctions.

## Results

### SplitFusion algorithm design

**Figure 1:**
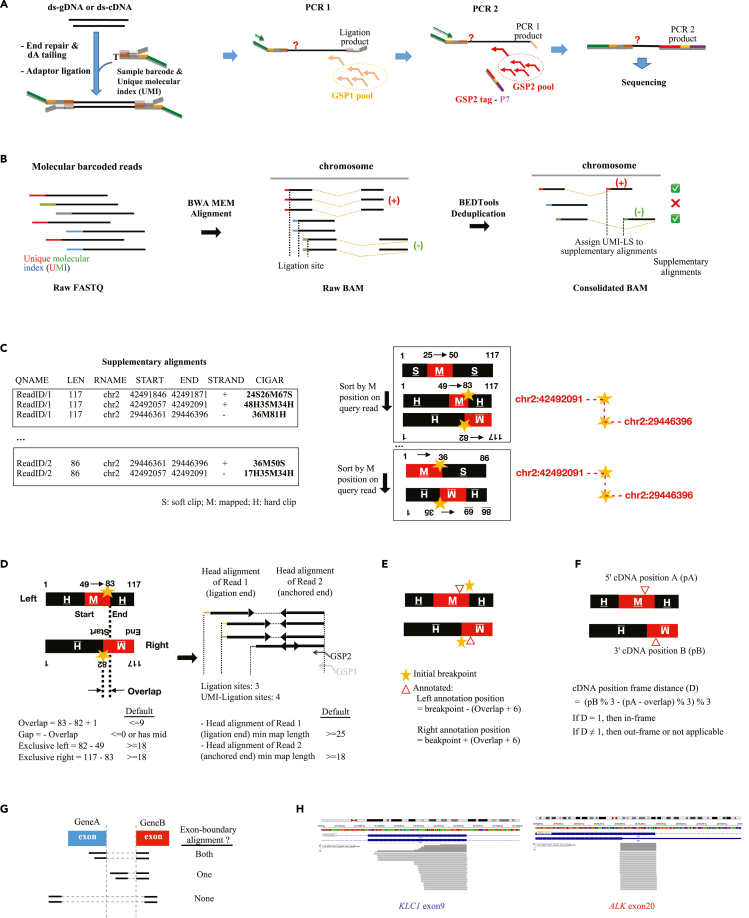
The framework of SplitFusion algorithm design (A) NGS library construction using the AMP. Double-stranded cDNA and genomic DNA (DNA) were subjected to end repair, dA tailing, and ligation of a half-functional Y adaptor, which contains a sample barcode and unique molecular identifier (UMI). Two rounds of hemi-nested multiplex PCR reactions were used to enrich any sequences (denoted by red question marks) downstream of the gene-specific primers (GSPs), resulting in an NGS library for sequencing. (B) NGS FASTQ data were de-multiplexed by sample barcodes followed by adaptor trimming. Different colors represent different UMIs tagged to different molecules. The data were aligned to a reference using BWA-MEM and de-duplicated based on a combination of a unique UMI and LS (UMI-LS). SAs were assigned with the same UMI-LS as their read 1 starting alignments. Reads with supplementary alignments (SAs) were extracted. (C) The CIGARs of reads with SAs were transformed. For the same read ID, all alignments were sorted by their mapping positions (M) on the query read, and the candidate fusion junction was inferred. (D–H) The initial candidate fusion junctions were filtered by maximum overlapping length, maximum gap, minimum exclusive mapping lengths of partner alignments, and the minimum mapping lengths of ligation end and anchored end alignments (D) and further subjected to gene annotation (E), frame-ness calculation (F), exon-boundary alignment judgment (G), and reporting and visualization (H).

SplitFusion can use both regular RNA-seq and anchored multiplex targeted enrichment like AMP (Figure 1A) NGS data. SplitFusion consists of the following major computation steps (the parameters are adjustable in the software, with the default values being arbitrarily based on our clinical experience accumulated since the AMP publication^18^).

#### Reference alignment and deduplication

SplitFusion leverages the fast and widely used genome mapping tool BWA-MEM,^19^ which generates supplementary alignments (the supplementary alignment [SA] tag in the sequence alignment map [SAM] specification represents other canonical alignments in a chimeric alignment) for a query sequencing read containing two or more parts mapped to different locations of the reference; i.e., multiple alignments (Figure 1B). When the genome (instead of the transcriptome) is used as the reference for RNA-seq reads, the different segments of a sequencing read can be aligned to different genes, neighboring exons, or sequences containing cryptic splice sites. SplitFusion recognizes FASTQ or binary alignment map (BAM) files in the specified directory as input. If the BAM file (BWA-MEM aligned) exists, then the kickstart mode will be initiated, bypassing the FASTQ-to-BAM reference alignment step. Then, deduplication and consolidation of the BAM is performed based on a combination of a unique molecular identifier (UMI) and the aligned chromosomal position of the ligation site (LS) of a DNA fragment. A single UMI-LS read (with all of its split alignments) will be kept to represent the reads with duplicated UMI-LSs, resulting in a consolidated BAM file (Figure 1B).

#### CIGAR string transformation

SplitFusion parses the Compact Idiosyncratic Gapped Alignment Report (CIGAR) strings and then orients and sorts the chimeric parts (split reads) by the order of their segments in the query read (Figure 1C). To decipher the alignment construct of a query read, the CIGAR string is first parsed into soft-clipped (S; unmapped but not trimmed), mapped (M), and hard-clipped (H; unmapped and trimmed) segments and their lengths. Short insertions and deletions (1 deletion/1 insertion) in the CIGAR string are removed, and the M segment length is adjusted accordingly. SplitFusion then uses these S/M/H lengths of all split alignments from the same read, taking into account their respective alignment orientations (plus or minus strand), and the query read lengths (as available from BEDTools^20^) to calculate the query read start and end positions of the different split alignments. SplitFusion uses the mapping chromosomal position of the LSs for detecting deduplicated reads (i.e., reads that share the same UMI-LSs) and for reporting the final number of partner end positions. However, for cDNA fragments, the heading sequences for adapter ligation are often partial exonic sequences (followed by coding sequences of their upstream/downstream exons) and are too short to be mapped to the reference genome. SplitFusion uses the lengths of these unmapped sequences (soft-clipped length) to adjust the LS mapping position (encoded in the read ID field) for accurate calculation of the number of partner ends later (LSs). SplitFusion then sorts all split alignments from the same query read by their original segment orders on the query read.

#### Candidate fusion junction detection

From the transformed split-read alignments, SplitFusion infers fusion junction candidates. For any two neighboring alignments on the same query read if they align to different chromosomes or the same chromosome but distant at >750,000 bases (the largest intron size used by the BLAST-like alignment tool [BLAT]).^21^ In the case of genuine fusion genes being very close (<750,000 bases; one can use the “SplitFusion-Target” model to call any such fusions), they are deemed candidate fusion partners and output to separate files (left and right). The partners are then merged by their read IDs, and their junction-corresponding chromosomal alignment positions are deemed candidate fusion junctions (Figure 1C). For a typical two-split alignment read, the merged new record has the construct “left query start – left query end [fusion junction 1] --- right query start [fusion junction 2] – right query end.” For a read that consists of three or more alignments (as long as the length of sequencing read can accommodate), such as those involving intra-gene neighboring exons of fusion partner genes, the leftmost and rightmost alignments are merged, and the new record has the construct “leftmost query start – nearest end to junction [fusion junction 1] --- nearest start to junction [fusion junction 2] – rightmost query end.” The middle alignments between the leftmost and rightmost are output to a separate file (“mid”) for downstream analyses.

#### Initial fusion junction filtering

As an initial step to minimize false positive results, SplitFusion filters the candidate fusion junctions by their minimum alignment length, minimum non-overlapping length of split parts, maximum gap length, and minimal number of LSs (UMI-LSs) (Figure 1D). SplitFusion can apply different minimum mapping length cutoffs on different partner alignments. Typically, for anchored end NGS data, this feature allows for high fusion detection specificity by specifying a high mapping length cutoff value (default 25; Figure 1D) on the ligation end and for high fusion detection sensitivity by specifying a short mapping length cutoff (default 18) on the anchored end. Additional specificity on the anchored end was already imposed in the wet lab protocol by target enrichment using outer gene-specific primers (GSP1s; Figure 1A), which were, however, not present in NGS data, because the sequencing primer is linked with the inner gene-specific primers (GSP2-tag P7; Figure 1A) so that the portions of the target sequences from the outer primers to the 5′ end of the inner primers are not sequenced but have contributed to the experimental specificity for the target.

#### Fusion junction gene annotation, frame status, exon boundary, further filtering, and target reporting

Next, SplitFusion annotates candidate fusion junctions with gene names, exon numbers, and cDNA positions (Figure 1E). It is common that split alignments of the same query read share a few identical ending DNA bases, which are typically double counted because they belong both to the exon of gene A and the intron of the partner gene B (adjacent to the fusion exon of gene B), largely due to canonical splice junction sequences.^22^ These double-counted overlapping alignment segments (which are the results of BWA alignment seed-extension searching) need to be accounted for in order to accurately calculate the fusion frame status. SplitFusion tackles this issue by temporally shifting the fusion junction positions by 6 bases (default) inward to the center of the alignments, for both of the involved split alignments, in order to annotate the fusion junctions with the correct cDNA positions and exon number when using annotation tools such as ANNOVAR^23^ or SnpEff.^24^ The middle alignments, if they exist, are also annotated. If a target gene panel browser extensible data (BED) file is specified in the initial configuration, at the beginning of this step, the “Target” mode will be employed, and only alignments involving the targeted genes, together with any potential partners, will be annotated. Following annotation, SplitFusion infers the frame status of the fusion transcript (in frame/out of frame or not applicable; Figure 1F) and judges whether the fusion junctions are on known exon boundaries (both/one/none) according to RefSeq^25^ (Figure 1G). Optionally, a backend “whitelist” database can be used for the targeted output to report alternative splicing (e.g., for *MET* exon 14 skipping), exon deletion (e.g., *EGFR vIII* exon 2–7 deletion), and gene truncation (e.g., *FGFR1/2/3* exon 18 truncation) as well as a “blacklist” database to remove recurring false positive detections. By default, when both fusion junctions are at exon boundaries/junctions, the minimum number of fusion partner ends (ligation ends) required to call a fusion is 1. When only one fusion junction is at an exon boundary, the minimum number of fusion partner ends (ligation ends) required to call a fusion is 3.

#### Detection in highly repetitive regions and pseudogenes

Highly repetitive regions, such as regions with homopolymer or pseudogenes, represent technical challenges during mapping to the reference genome; for example, the DUX4 region consists of 11 to >100 repeated segments, each of which is about 3.3 kb long and contains one copy of *DUX4*, while the other copies are described as “*DUX4-*like” [*DUX4L*].^26^ However, these regions could be clinically significant, such as the DUX4 region, which is involved in *CIC*::*DUX4* fusion-positive sarcomas. To address this issue, SplitFusion uses a user-specified BED file to explicitly handle fusions involving partners in highly repetitive regions. BWA-MEM assigns alignments on repetitive regions a mapping quality of 0, and alternative alignment coordinates are also recorded (in the XA:Z field). SplitFusion checks an alignment with a mapping quality of 0 and its alternative alignments against a user-specified BED file (pseudogene.bed) and, if overlapped, its alignment coordinates are then updated to the coordinates of the specified region. As a result, highly repetitive sequences are mapped to the user-specified regions for increased sensitivity in detecting fusions.

#### Result reporting and visualization

SplitFusion outputs a final fusion summary, including exact fusion junctions, number of unique supporting reads (according to UMI), number of partner ends (i.e., LSs), frame status, exon-boundary status, fusion gene names (with transcript IDs) and exon numbers. Last, if a fusion is detected, 10 fusion-supporting split reads (in FASTQ) will be randomly extracted from the consolidated BAM file, saved as a text file that can be examined manually, and converted into a BAM file that can be visualized with Integrated Genome Viewer (IGV)^27^ or a third-party software (Figure 1H).

### Benchmarking performance versus other tools

**Figure 2:**
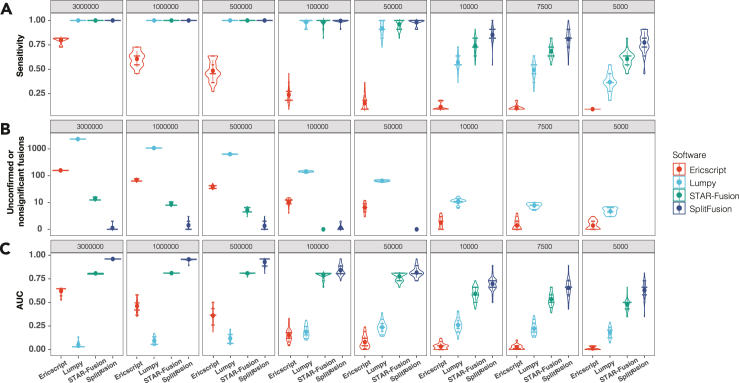
Benchmarking the sensitivity, specificity, and accuracy of EricScript, Lumpy, STAR-Fusion, and SplitFusion The datasets of the 11 positive samples were randomly down-sampled to different sizes (reads: 3M, 1M, 500K, 100K, 50K, 10K, 7.5K, and 5K), each size with 21 replicates (random seeds), generating 1,848 datasets for analyses by the four tools with their default parameters using one computation thread. (A) Sensitivity was calculated as the fraction of samples resulting in expected gene fusions (*ALK/EWSR1*) among the 11 samples of a given size and given random seeds. (B) Specificity was evaluated by the number of unconfirmed or non-significant fusions; i.e., other than *ALK* and *EWSR1*. (C) The overall accuracy was calculated by the area under the precision-recall curve (AUC) at varied supporting read thresholds (from minimum to maximum supporting reads) according to a previous method. The violins outline the distribution (the solid circle is the mean) of the statistics for each group (21 datasets).

To evaluate the performance of SplitFusion, we tested 46 cancer cases with known clinical diagnoses. FFPE tissue sections from 10 *ALK* fusion-positive lung cancers, 1 *EWSR1* fusion-positive sarcoma, and 35 *ALK* fusion-negative lung cancer cases (as confirmed by the routine fluorescence *in situ* hybridization [FISH] assay; Figure S1) were subjected to a 19-gene lung panel (Table S1) targeted RNA-seq assay using AMP. We compared four fusion detection tools: EricScript,^14^ Lumpy,^15^ STAR-Fusion,^16^ and SplitFusion. To test how the sensitivity depends on coverage, we randomly down-sampled 11 fusion-positive sample datasets to different numbers of reads (3M, 1M, 500K, 100K, 50K, 10K, 7.5K, and 5K read pairs), with 21 replicates of each size, generating a total of 1,848 datasets that were analyzed using the four fusion detection tools (Figure 2A). With 500K or more reads, nearly all tools showed a sensitivity of 100% (231 of 231 for all 3 sizes ≥500K), with the exception of EricScript (average sensitivity of 45% for 500K reads; Figure 2A). The tools had varied levels of additional fusions detected that were not reported previously for these samples (For 500K reads, EricScript and Lumpy had >100 additional fusions per dataset, as they do not include filtering steps by default; STAR-Fusion, 5–14; SplitFusion, 2–3; Figure 2B). Gradually reducing the size of datasets, the sensitivities of the tools started to differentiate (at 10K read: EricScript 11% [25 of 231], Lumpy 57% [132 of 231], STAR-Fusion 74% [171 of 231], SplitFusion 85% [196 of 231]; at 5K read: EricScript 9% [21 of 231], Lumpy 37% [85 of 231], STAR-Fusion 61% [141 of 231], SplitFusion 78% [180 of 231]), and the number of still unreported fusions was lowest for STAR-Fusion and SplitFusion (Figure 2B). We assess the overall accuracy of the different methods by calculating the precision-recall area under the curve (AUC)^16^ by varying the number of reads that are required to support a fusion. SplitFusion showed the highest AUCs among the tools across all different dataset sizes (Figure 2C). Indeed, in the smallest 5K-read datasets, the mean AUCs for EricScript, Lumpy, STAR-Fusion, and SplitFusion were 0.01, 0.18, 0.48, and 0.63, respectively. As for computing resources, STAR-Fusion requires ∼30 GB RAM, which is much higher than that of Lumpy (∼10 GB) and EricScript and SplitFusion (both at ∼4 GB; a typical computation cluster configuration: 4 GB random-access memory [RAM] per thread) (Figure S2A). For the largest 3M-read datasets, the computation times were 0.65, 0.51, 0.46, and 0.42 h for EricScript, Lumpy, STAR-Fusion, and SplitFusion, respectively (Figure S2B). It is worth noting that both STAR-Fusion and SplitFusion have kickstart modes that can skip reference mapping and start from BAM, which reduces user time by >50% (Figure S2B).

**Figure 3:**
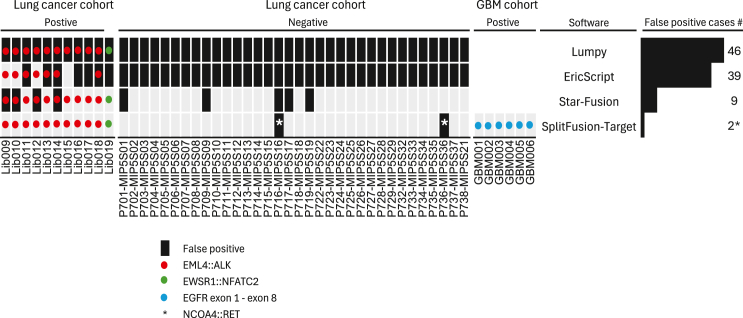
Fusion detection results for the 46 lung cancer samples with known clinical diagnoses (11 positive and 35 *ALK* negative) by EricScript, Lumpy STAR-Fusion, and SplitFusion Each column represents one sample. Different colors and symbols denote different fusions. The numbers of total false positive calls (other than *ALK* or *EWSR1*) for each software are plotted in the bar plot. Of 35 cases of glioblastoma (GBM), six were found with EGFR vIII using SplitFusion-Target. The GBM samples were not subject to analysis by the other three software packages.

Among the 35 *ALK* fusion-negative lung cancer samples, EricScript and Lumpy reported (by default without filtering) 39 and 46 unknown-significance fusions, respectively, whereas STAR-Fusion reported five such fusions involving the tumor suppressor gene *CDKN2A*, and SplitFusion reported two *NCOA4::RET* cases with the *NCOA4* fusion junction aligned on the exon boundary (Figures 3 and S3).

### Validation using TCGA RNA-seq data

Using the TCGA RNA-seq data from 515 patients with lung adenocarcinoma (LUAD), we compared the performance of SplitFusion with three previous studies, STAR-Fusion,^28^ TumorFusions,^29^ and FusionGDB.^10^ For the purpose of detecting clinically relevant kinase fusion, we focused on fusions involving genes manually curated previously^28^ and with clear clinical significances^18^ in LUAD; namely, *ALK*, *ROS1*, *RET*, *NRG1*, *NTRK1/2/3*, *BRAF*, *MET*, *PRKCB*, *RPS6KB1* (as 3′ partners), and *FGFR1/2/3* (as a 5′ partner). Of the 22 cases with the recurrent gene fusions detected by any of the three previous studies, STAR-Fusion detected 17 (of 21; one case not available), TumorFusions detected 19, FusionGDB detected 19, while SplitFusion detected all 22. Of the remaining 493 LUAD cases, the three studies showed no fusions involving any of the recurrent genes, while SplitFusion detected two cases with *SLC34A2::ROS1* fusion (Tables 1 and S4; Figure S9), an indication for ROS1-targeted therapy in lung cancer patients. Visual inspection of the output produced by SplitFusion confirms these two cases (albeit with low read counts) with high confidence because the co-existing variants spanned different partner exons, as we observed routinely in the clinic.^18^ Among all fusions detected, two samples involved 5′ UTR partners, one a 5′ partner, and one a 3′ partner (Figure S10). For an example case of the SplitFusion output, please see Table S5.

**Table 1:** Comparisons of calling recurrent gene fusions in TCGA LUAD samples by STAR-Fusion, TumorFusions, FusionGDB, and SplitFusion

Recurrent fusion^a^	STAR-Fusion		TumorFusions		FusionGDB		SplitFusion	
	+	–	+	–	+	–	+	–
Positive (*n* = 22)	17	4	19	3	19	3	22	0
Negative (*n* = 493)	0	467	0	493	0	492	2^b^	491

See Table S4 for detailed fusion call lists.

a Recurrent gene fusions are defined as those involving genes manually curated previously in LUAD (*ALK*, *ROS1*, *RET*, NRG1, *NTRK1/2/3*, *BRAF*, *MET*, *PRKCB*, and *RPS6KB1* as 3′ partners and *FGFR1/2/3* as 5′ partners) and called by any of the three previous studies (STAR-Fusion, Tumor Fusions, and FusionGDB).

b Two samples with *SLC34A2::ROS1* fusions were only called by SplitFusion, confirmed with IGV visualization of the reads (Figure S9).

### SplitFusion targeted output

With the Target mode, SplitFusion can report targeted outputs, such as specific alternative splicing, exon skipping, and deletions, as well as filter recurrent false positives. To demonstrate this feature, we specified *EGFR vIII* (exons 2–7 deletion), a known oncogenic driver in glioblastoma, in the target output file when analyzing FFPE samples from 35 glioblastoma cases (mean age 54.8 years, male 65.7%). SplitFusion-Target reported six *EGFR vIII* cases (Figures 3 and S4). To test detecting fusions involving unannotated cryptic exons, we tested two TCGA prostate adenocarcinoma samples with known *ARv7* variants.^30^ SplitFusion-Target accurately detected the variants in both cases (Figure S5), whereas none of the 515 TCGA LUAD samples were positive.

### Expanded clinical analyses in 1,076 lung cancer FFPE samples

**Figure 4:**
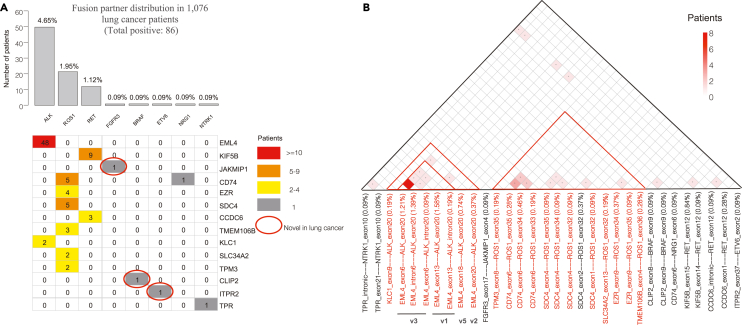
The analysis of 1,076 clinical lung cancer FFPE samples using SplitFusion (A) The prevalence of different target gene fusions is shown in the bar plot. In total, 86 (8.0%) samples were fusion positive. The numbers of target genes with partner genes (right column) are indicated in the cells. (B) The numbers of co-occurrences of fusions in the same samples are plotted in the matrix. The *EML4-ALK* fusion variants are denoted under each specific fusion.

To evaluate the robustness of SplitFusion performance in clinical samples, we expanded SplitFusion analyses on targeted sequencing of 1,076 clinical lung cancer (mean age 59.6 years, male 57.8%) FFPE samples using a 62-gene panel (Table S2). SplitFusion detected 86 (8.0%) fusion-positive cases in lung cancers (Figure 4A). The prevalence of *ALK* (4.65%), diverse partner::*ROS1* (1.95%), and diverse partner::*RET* (1.12%) fusions was comparable to previously reported frequencies.^31^ Rare oncogenic drivers and therapeutically relevant gene fusions (*KLC1::ALK*, *CD74::NRG1*, and *TPR::NTRK1*) were also identified (Figures 4A and S6). Interestingly, SplitFusion detected novel fusions that have not been reported previously in lung cancer, including one squamous cell carcinoma with *FGFR3::JAKMP1* fusion, one adenocarcinoma with *CLIP2::BRAF* fusions (two fusion isoforms in one tumor), and one adenocarcinoma with *ITPR2::ETV6* fusion (Figures 4A and S7).

Multiple gene fusion variants can co-occur in the same tumor,^18^ and different variants may affect clinical outcomes.^32^^,^^33^^,^^34^ However, the co-occurrence frequency of different fusions is largely unknown. Using SplitFusion, we found that multiple gene fusion variants with in-frame exons of partner genes, with or without involving cryptic splice site sequences, co-occurred frequently in the same tumors (Figure 4B). Among the kinase genes analyzed, *EML4::ALK* v3- and *CD74::ROS1-*harboring lung tumors had the most frequent co-occurrences of multiple fusion variants.

#### Fusion junction-defined subclone

**Figure 5:**
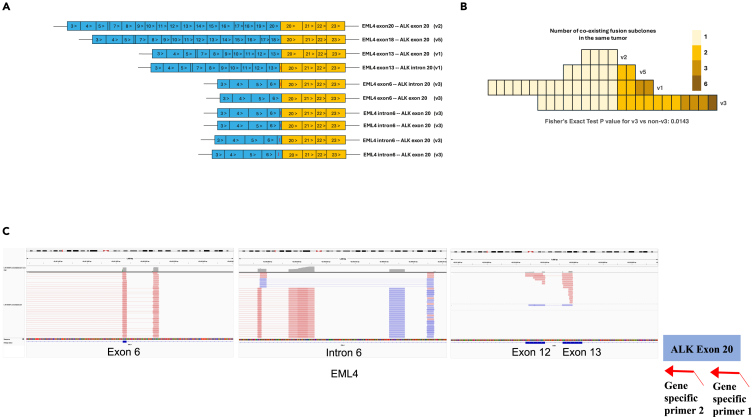
The analysis of fusion junction-defined subclones in *EML4-ALK*-positive lung tumors (A) Schematic representations of different *EML4-ALK* fusion variants. Blue cells represent *EML4* exons, and yellow cells represent *ALK* exons. Numbers inside the cells show exon numbers. Cells with red strokes represent introns. (B) Each tile represents one patient. The tile color gradients denote the number of co-existing fusion subclones, defined by exact fusion junction, in the same tumor. Fisher’s exact test was performed to compare coexisting fusion variants in the same tumor (1 vs. ≥2) by variant type (v3 vs. non-v3). (C) For one *EML4-ALK* v3 lung tumor sample, the Integrated Genome Viewer (IGV) showed 6 fusion junctions in *EML4* exon 6, intron 6, and exon 13. Their gene partner *ALK* exon 20 is schematically shown on the right in red and with nested gene specific primers 1 and 2.

To dissect the intratumor clonal heterogeneity, fusion subclones were defined by the exact fusion junctions inferred from the genomic mapping of fusion RNA sequences (Figure 5A). Our results showed that 55% of *EML4::ALK* v3 tumors (11 of 20) had co-existing heterogeneous fusion subclones in the same tumors (7 two-subclone tumors, 3 three-subclone tumors, and 1 six-subclone tumor; Figures 5B and 5C), which was significantly higher than 19% (6 of 31) in other variant tumors (4 of 19 v1, 2 of 8 v5, and none of 4 v2; Fisher’s exact test, two-sided, *p* = 0.0143).

### Fusions involving highly repetitive sequences or pseudogenes

To evaluate the SplitFusion algorithm in detecting highly repetitive genes, we used two clinical sarcoma cases (Massachesetts General Hospital [MGH] case #1 and case #2) with confirmed *CIC::DUX4* fusion, detected using a clinically validated assay based on AMP targeted RNA-seq. The results showed that, in both cases, *CIC::DUX4* were correctly detected by SplitFusion (Figure S8). While case 1 had one dominant in-frame fusion, case 2 had multiple in-frame fusion variants (Table S3).

## Discussion

We demonstrate that SplitFusion is fast, highly sensitive, specific, and computationally efficient in detecting gene fusions. SplitFusion can infer fusion frame status and exon-boundary alignment, which are particularly desirable in the clinical setting to assist in automating the non-trivial filtering of false positives or functionally insignificant fusion candidates. SplitFusion Target mode allows for continuous evidence-based improvement in clinical reporting. Challenging fusions involving repetitive regions can be sensitively detected using SplitFusion Pseudogene mode. Furthermore, SplitFusion is agnostic to known coding transcripts and can profile multiple co-existing fusion subclones, providing a powerful tool for research studies. Validation using the TCGA data shows superior sensitivity of the SplitFusion algorithm, and, especially, it performs well even with the short 48 bp TCGA RNA-seq data.

In general, a shorter-mapping-length filter increases detection sensitivity and decreases specificity compared to a longer one. Setting the length parameter would be a trade-off and may depend on different scenarios. For example, analyzing high-quality data generated from RNA obtained from fresh-frozen samples may benefit from using a longer length to increase specificity, whereas analyzing RNA from FFPE samples may benefit from using a shorter length to increase sensitivity. While normalization fusion calls by fragment per million may be helpful in improving fusion detection, SplitFusion has not attempted to normalize fusion calls at the entire gene level. Instead, SplitFusion focuses on the fusion junction (parameters: minPartnerEnds_BothExonJunction and minPartnerEnds_OneExonJunction), as we are interested in the presence or absence of a gene fusion regardless of the RNA expression level (low-expression genes such as *ALK* in lung tissue and high-expression genes such as *ROS1*) or the subclone fraction. One unique feature of SplitFusion is its reliance on fusion junctions on an exon boundary, allowing the requirement of only one single supporting read to call a gene fusion when both partner junctions are on exon boundaries and three supporting reads when only one junction is on an exon boundary without compromising specificity and, in return, maximizing sensitivity. However, for assay quality control (QC), one can use the minimal number of RNA reads from housekeeping genes as the indicator for QC (passed/failed).^18^

Limitations of SplitFusion include the inability to detect non-reference sequences as fusion partners, such as from viruses. In this case, one can combine human and suspected viral genome FASTA files and use BWA (“bwa index”) to build an extended reference genome. By design, SplitFusion relies on at least one split read supporting a fusion between two partners. Some tools allow detecting fusion based on paired-end supporting reads only; namely, read 1 solely aligned on gene A and read 2 solely aligned on gene B. However, without the support of a split read crossing the fusion junction, inference of frame status, an important feature in fusion characterization, would not be possible conclusively. The kickstart mode of SplitFusion requires a BAM file from an aligner that generates split reads, with the first 10 standard fields in the SAM format and two additional fields, SA and XA, as in the standard SAM specification. We have not yet tested whether non-BWA-MEM aligned BAM files are compatible with SplitFusion.

In a previous matched tissue-blood analysis, 21% of ALK-positive non-small cell lung cancer patients showed multiple tumor-specific *EML4::ALK* variants,^35^ indicating the existence of fusion-defined tumor heterogeneity evident at the genomic level. SplitFusion can comprehensively map cryptic splice-site fusion junctions and has identified up to six subclones co-existing in one *EML4::ALK* v3 tumor with different fusion junctions in *EML4* intron 6. Increasing evidence suggests that lung cancer patients with *EML4::ALK* v3 tumors may have less favorable clinical outcomes compared to patients with other *EML4::ALK* variants.^34^^,^^36^ Consistent with these observations, we recently used SplitFusion and further demonstrated that intratumoral *EML4::ALK* isoforms may have different sensitivities to ALK inhibitors and may predict the efficacy of targeted therapy in *ALK*-rearranged lung cancer.^37^ Among *ROS1*-positive lung cancers, we also observed that *CD74::ROS1* tumors had more fusion subclones than *ROS1* tumors with other fusion partners. Interestingly, one recent study showed that lung cancer patients with *CD74::ROS1* had a lower survival rate compared to those with other *ROS1* partners.^38^ Together, these findings suggest that fusion-defined subclone analyses may be important in refining diagnosis and treatment for fusion-positive tumors in the future.

Our observation of the fusion junction hot zone in *EML4* intron 6 needs to be confirmed by larger studies. Further, whether this phenomenon could explain the resistance to the targeted treatment of the *EML4::ALK* v3 tumors requires further mechanism studies and prospective clinical investigations. In conclusion, SplitFusion is fast, sensitive, specific, and well suited for broad use in clinics for gene fusion detection as well as in research for studying fusion-defined tumor heterogeneity.

In conclusion, SplitFusion is a fast, open-source tool suitable for clinical-grade fusion detection. SplitFusion demonstrated superior benchmark performance in FFPE samples from clinically diagnosed cases compared with current well-established tools, with higher sensitivity, specificity, and minimal computation resources. In addition to targeted (anchored) RNA-seq data, SplitFusion also adapts to regular RNA-seq data, like TCGA short-read data, and features highly desirable abilities for clinical reporting, including frame status inferring, exon-boundary alignment, and targeted alternative splicing reporting. SplitFusion outperformed others in challenging scenarios, such as detecting highly repetitive regions like *DUX4*. Furthermore, SplitFusion is suitable for studying fusion-defined tumor heterogeneity.

## Methods

### Performance benchmarking with other tools

We selected three other fusion detection tools to benchmark the performance of SplitFusion for several reasons. In a recent comprehensive comparison of 23 different tools, STAR-Fusion was among the most accurate and fast tools.^16^ EricScript^14^ was top-ranked among 12 well-known tools in an earlier study.^39^ Lumpy was not among the tools compared by the aforementioned two studies but was able to integrate the read pair, split read, and read depth jointly,^15^ especially when the structural variation (fusion) signal is reduced, owing to either low-coverage data or low intra-sample fusion variant allele frequency, which are common in cancer samples.

Performance of the four tools was compared using 46 RNA-seq datasets generated from clinical FFPE samples (11 positive and 35 negative cancer cases; see Clinical NGS for details). For the 11 positive sample datasets, each was randomly down-sampled using the seqtk tool (v.1.0-r31; https://github.com/lh3/seqtk) to eight different sizes (3M, 1M, 500K, 100K, 50K, 10K, 7.5K, and 5K read pairs); each size had 21 replicates, generating a total of 1,848 datasets for the benchmarking test. All tools were run with one computing thread using the human genome GRCh37-hg19 as the reference, and fusion analyses were run using default parameters (e.g., the default threshold for STAR-Fusion to detect a fusion is one split-read [min_junction_reads default: 1]).

The performance of the four tools was scored based on the following metrics: (1) Sensitivity—the fraction of samples with the correct fusion genes (*ALK* and *EWSR1*) detected among the 11 fusion-positive samples (2) Previously unreported fusions—fusions involving genes other than the expected *ALK* and *EWSR1*; (3) AUC—the area under the precision-recall curve, generated by varying the threshold for the number of supporting reads (from minimum to maximum supporting reads) according to the previous method^16^; (4) peak memory—the maximum resident set memory size (the portion of memory occupied by a process that is held in main memory) during running of the tool; and (5) user time—the amount of central processing unit (CPU) time for a given analysis. Publicly available database and tool versions used were as follows: human genome reference hg19, Lumpy v.0.2.13, EricScript v.2.1, STAR-Fusion v.1.5, bwa v.0.7.17, Samtools v.1.10, BEDTools v.2.27.1, seqtk v.1.0-r31, ANNOVAR (October 24, 2019, PERL v.5, R v.3.6.3, and Python v.2.7/v.3.

### Clinical NGS

In this study, 10 *ALK*-positive lung cancer samples and one *EWSR1*-positive sarcoma^40^ sample, confirmed by clinical FISH assays, and 35 *ALK* FISH-negative lung cancer samples from Queen Elizabeth Hospital, Hong Kong, were included. Total nucleic acids (TNA) containing RNA and DNA were extracted from FFPE sections using the Agencourt FormaPure Kit for FFPE Tissue (Beckman Coulter, Indianapolis, IN, USA). NGS libraries were constructed using the AMP targeted RNA-seq method described previously,^18^ with modifications for incorporating UMIs in the adapters and using a one-tube assay for simultaneous enrichment of RNA and DNA targets.^41^ Briefly, 100 ng of TNA was used for reverse transcription by SuperScript IV (Thermo Fisher Scientific) using random hexamers, followed by second-strand synthesis. The double-strand cDNAs (ds-cDNAs) were purified using 1.8× SPRIselect (Beckman Coulter) and subjected to further library construction using the KAPA HyperPlus Kit (Roche). The purified ds-cDNAs were enzymatically fragmented at 37°C for 15 min and then end repaired and A tailed to produce 5′-phosphorylated and 3′-dA-tailed by incubating with the end-repair and A-tailing enzyme at 37°C for 15 min, followed by 65°C for 15 min. Adapters containing a sample index and UMI^42^ were then ligated to the end-repaired and A-tailed DNA fragments using DNA ligase with 25 pmol of annealed adapters at 16°C for 30 min followed by 22°C for 30 min. Post-ligation cleanup was performed using 1.0× SPRIselect (Beckman Coulter). The size-selected ligation products were eluted in 20 μL nuclease-free water and amplified by two rounds of hemi-nested PCRs for target enrichment. In PCR1, the ligated DNA fragments were added to 3 μL of 10× PCR buffer with a final concentration of 2 mM of MgCl_2_, 0.2 mM of deoxynucleotide triphosphate (dNTP) (New England Biolabs), 25 pmol of panel-specific GSP1 enrichment primers, and 3 units of Platinum Taq DNA polymerase (Invitrogen) and topped up to a final volume of 30 μL of nuclease-free water. The thermal cycling conditions were as follows: initial denaturation at 95°C for 5 min followed by 20 cycles of 95°C for 30 s and ramping down at −0.2°C/sec to 60°C for 1 min; hold at 4°C. The amplified PCR1 products were washed with 1.2× SPRIselect and eluted in 20 μL nuclease-free water. In PCR2, 20 μL of the purified PCR1 products were mixed with 3 μL of 10× PCR buffer, a final concentration of 2 mM of MgCl_2_, 0.2 mM of dNTP, 25 pmol of panel-specific GSP2 enrichment primers, 10 pmol of P5 primer, 10 pmol of P7 indexing primer,^42^ and 3 units of Platinum Taq DNA polymerase and topped up to 30 μL with nuclease-free water. The thermal cycling parameters were as follows: initial denaturation at 95°C for 5 min followed by 15 cycles of 95°C for 30 s and 65°C for 5 min; hold at 4°C. The final library constructs were size selected by 0.7× SPRIselect and eluted in 30 μL of elution buffer. After quantification by KAPA Library Quantification Kit for Illumina (Roche), the constructed libraries were subsequently pooled and sequenced by the Illumina NextSeq 500 system. Similarly, the lung cancer and glioblastoma cohort samples were analyzed as described previously.^43^ The 1,073 expanded lung cancer cases were collected in Zhejiang Cancer Hospital, subjected to the same laboratory protocols, and sequenced to, on average, ∼3M sequencing reads per sample. The *ALK* fusion of the expanded lung cancer cases were initially diagnosed using immunohistochemistry (VENTANA ALK D5F3 CDx assay) or RT-PCR (AmoyDx *EML4-ALK* [2013/3400389]) clinical assays. *ROS1*, *RET*, and other rare fusions were detected by NGS analysis, and the fusion reads were confirmed by manual inspection with IGV.

### TCGA RNA-seq data analysis

To evaluate the performance of SplitFusion using RNA-seq data generated from methods other than the anchored targeted RNA-seq, we used the TCGA RNA-seq data via the Google Cloud-based Terra platform (https://app.terra.bio/). We developed a Docker image to run SplitFusion on the Terra platform (see the SplitFusion GitHub repository).

### The *CIC::DUX4* fusion sarcoma cases

To test the performance of SplitFusion in highly repetitive sequences, we performed AMP-based targeted RNA-seq on two sarcoma cases with *CIC*::*DUX4* fusions at the Massachusetts General Hospital Center for Integrated Diagnostics.

### Statistical analysis

All statistical comparisons are two sided.

## Resource availability

### Lead contact

The lead contact for this study is Zongli Zheng (Zongli.Zheng@cityu.edu.hk).

### Materials availability

This study did not generate new unique reagents.

### Data and code availability

The code implementing all computational steps is included in the SplitFusion package. SplitFusion is available on GitHub (https://github.com/Zheng-NGS-Lab/SplitFusion) and Figshare.^44^ A user manual and example datasets are also included.

## Acknowledgments

We thank all patients who contributed their samples to this study. We thank Oscar Leung for proofreading the manuscript. This work was supported by the Ming Wai Lau Centre of Reparative Medicine of Karolinska Institutet (Lau grant LC230003), the 10.13039/501100001809National Natural Science Foundation of China (81672098 to Z.Z. and 81872072 to J.C.), The Hong Kong Research Grants Council (11319516 to M.Y. and Z.Z. and 11103024 and T12-101/23-N to Z.Z.), The 10.13039/501100004359Swedish Research Council (202001418 to Z.Z.), Shenzhen Medical Research Fund (B2402002 to Z.Z.), the Project of Shanghai Municipal Science and Technology Commission (21Y31900301 to J.W.), and the 10.13039/501100017531Medical and Health Research Project of Zhejiang Province (WKJ-ZJ-2418). G.G. was partially funded by the Paul C. Zamecnik Chair in Oncology at the Massachusetts General Hospital Cancer Center. W.B. was partially supported by the 10.13039/501100004543China Scholarship Council (201806220003). We thank NCBI dbGaP for access to the TCGA data (NCI-INC0794464). The study using clinical FFPE samples was approved by the Research Ethics Committee, Hospital Authority, Hong Kong (KC/KE-16-0262/ER-1) and institutional review boards at Zhejiang Cancer Hospital (IRB-2018-24) and Shanghai Changzheng Hospital (2006LL022), China.

## Author contributions

W.B., B.Z., B.A.K., Y.M.C., and C.B. performed bioinformatics analyses, built a Terra workflow, and analyzed the TCGA data. Z.S., C.X., W.W., C.L., and Y.Z. designed and analyzed the large lung cancer cohort. A.H.Y.C. performed bioinformatics analysis using benchmarking tools. H.W., Z.G., and J.C. designed and analyzed the glioblastoma cohort. C.L., S.B., H.Y.K., and M.Z.-Y.C. performed NGS experiments. V.N. performed the sarcoma study. W.C., M.Y., and W.C.S.C. designed and analyzed the initial clinical cohort study. G.G., M.Y., W.C.S.C., J.W., J.C., and Z.Z. supervised the research and obtained funding. Z.Z. designed SplitFusion. W.B., B.Z., and Z.Z. wrote the paper. All authors approved the paper.

## Declaration of interests

Z.Z. receives patent royalty from ArcherDX, a licensee of the AMP technology from Massachusetts General Hospital. Z.Z. is a co-founder and advisor of and holds equity in GenEditBio, whose interests are reviewed and regulated by institutional Outside Practice policies annually. G.G. receives research funds from IBM, Pharmacyclics, and Ultima Genomics and is an inventor on patent applications related to MSMuTect, MSMutSig, MSIDetect, POLYSOLVER, SignatureAnalyzer-GPU, and MinimuMM-seq. G.G. is a founder and consultant of and holds privately held equity in Scorpion Therapeutics. G.G. received travel support from Caris Life Sciences.

## Supplemental information

Document S1. Figures S1–S10 and Tables S1 and S2

Table S3. Output results of SplitFusion calling in two MGH cases with *CIC::DUX4* fusions using a clinically validated AMP NGS assay

Table S4. Comparison of fusion detection results from three previous studies (STAR-Fusion, TumorFusions, and FusionGDB) and SplitFusion using the TCGA RNA-seq data from 515 patients with LUAD

Table S5. SplitFusion output of fusion calling result for the TCGA sample LUAD-44-2665

Document S2. Article plus supplemental information
