# Supplementary material for: SplitFusion enables ultrasensitive gene fusion detection and reveals fusion variant-associated tumor heterogeneity
Source: Patterns (N Y). 2025 Feb 14;6(2):101174. doi: 10.1016/j.patter.2025.101174 (PMC11873004; doi:10.1016/j.patter.2025.101174)
Supplement: Document S1. Figures S1–S10 and Tables S1 and S2 [file mmc1.pdf]

Patterns, Volume *6*

Supplemental information

SplitFusion enables ultrasensitive gene fusion detection and reveals fusion variant-associated tumor heterogeneity

Weiwei Bian, Baifeng Zhang, Zhengbo Song, Binyamin A. Knisbacher, Yee Man Chan, Chloe Bao, Chunwei Xu, Wenxian Wang, Athena Hoi Yee Chu, Chenyu Lu, Hongxian Wang, Siyu Bao, Zhenyu Gong, Hoi Yee Keung, Zi-Ying Maggie Chow, Yiping Zhang, Wah Cheuk, Gad Getz, Valentina Nardi, Mengsu Yang, William Chi Shing Cho, Jian Wang, Juxiang Chen, and Zongli Zheng

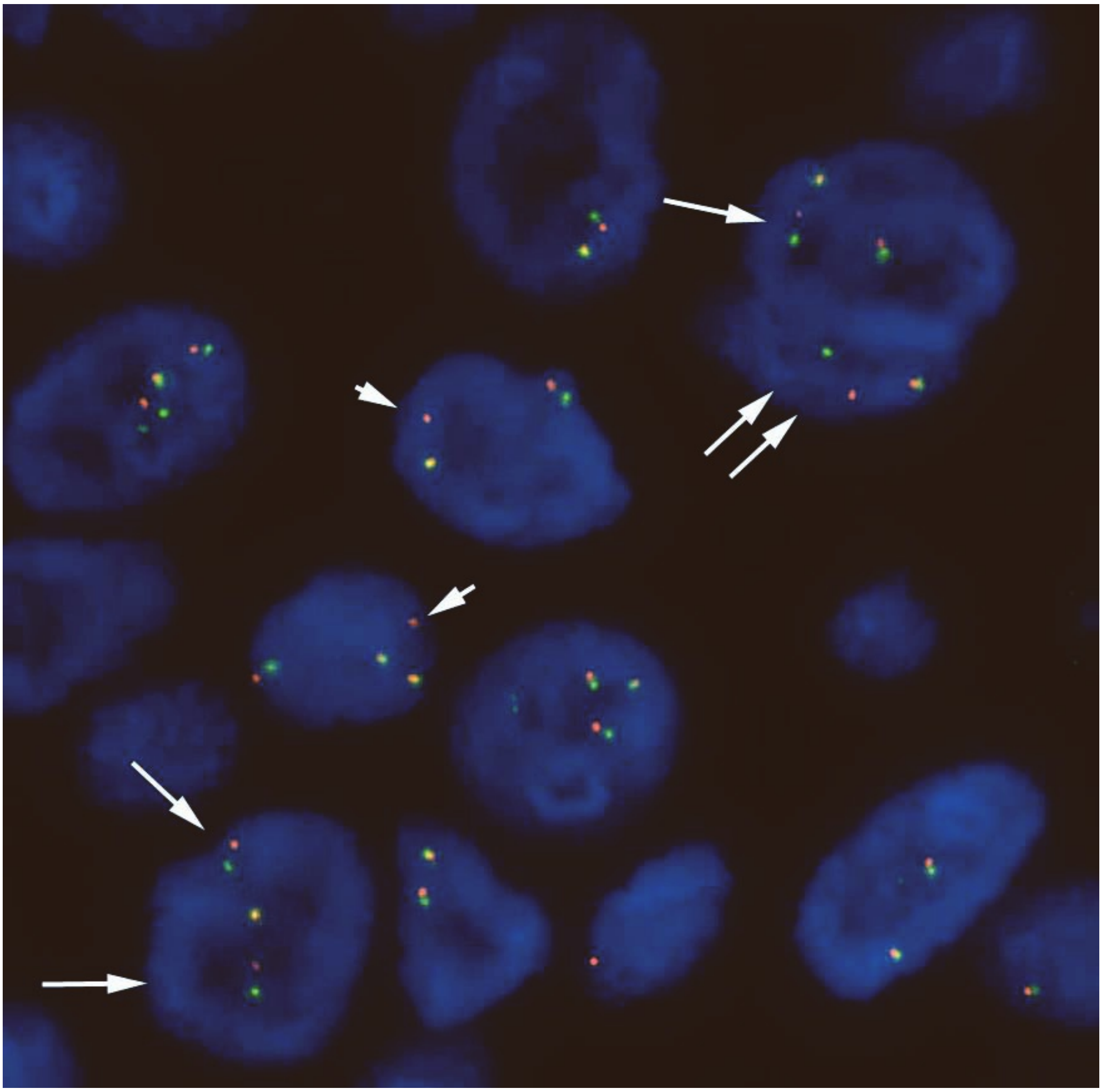

Figure S1. Fluorescence *in situ* hybridization (FISH) performed on a lung cancer formalin-fix and paraffin-embedded sample using Vysis ALK breakapart probes. The result indicated the presence of *ALK* gene translocation: separation of 3’ (red) and 5’ (green) signals were indicated by double arrows; narrow split of signals indicated by single arrows; isolated 3’ signals indicated by short arrows.

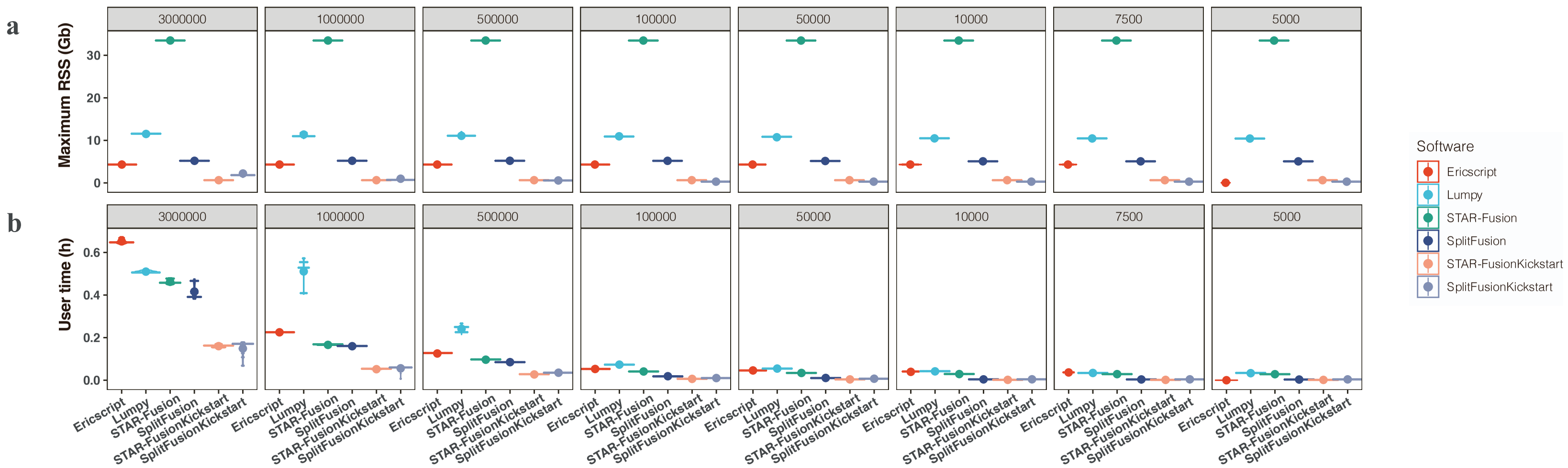

Figure S2. Benchmarking computation memory use and speed among the four tools – Ericscript, Lumpy, STAR-Fusion and SplitFusion. The datasets of the 11 positive samples were randomly down-sampled to different sizes (reads: 3M, 1M, 500K, 100K, 50K, 10K, 7.5K and 5K), each size with 21 replicates (random seeds), generating 1,848 datasets for analyses by the four tools with their default parameters using one computation thread. (**a**) Maximum RSS: the maximum resident set size (RSS: the portion of memory occupied by a process that is held in main memory) during the computation of the software. (**b**) User time: the amount of total CPU time for a given analysis.

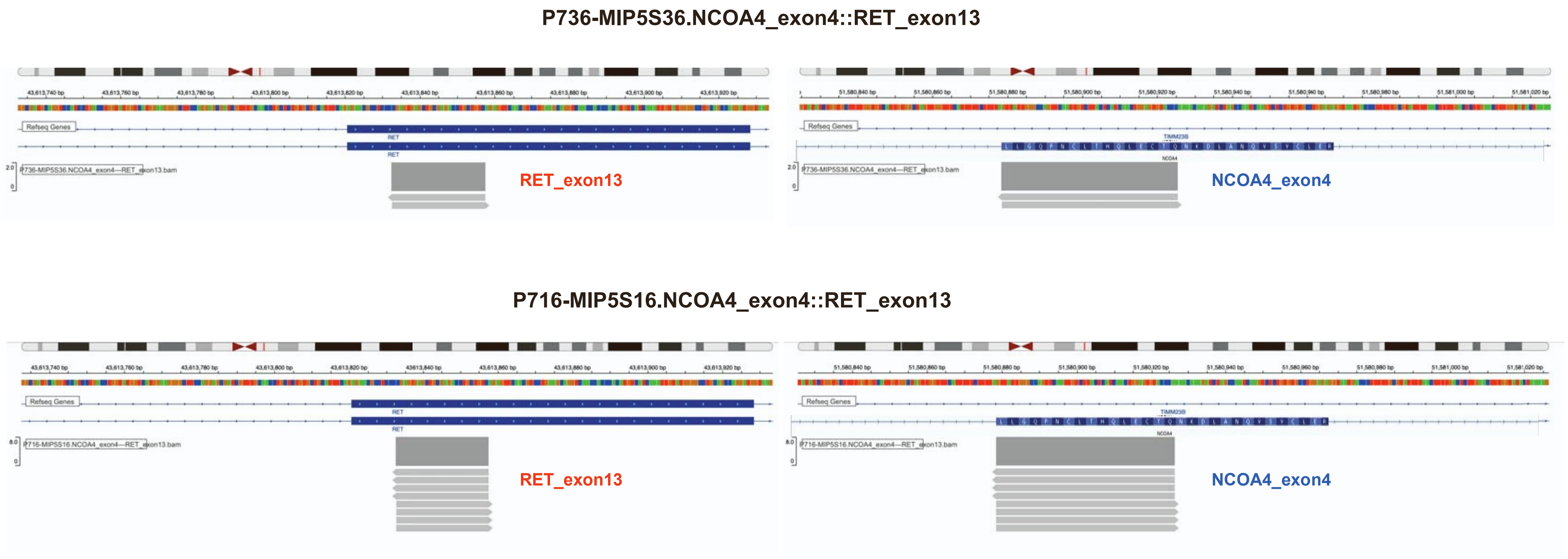

Figure S3. Visualization of *NCOA4-RET* gene fusions showed the alignment of detected partner gene *NCOA4* (colored in blue) on exon boundary.

EGFR exon1::EGFR exon8

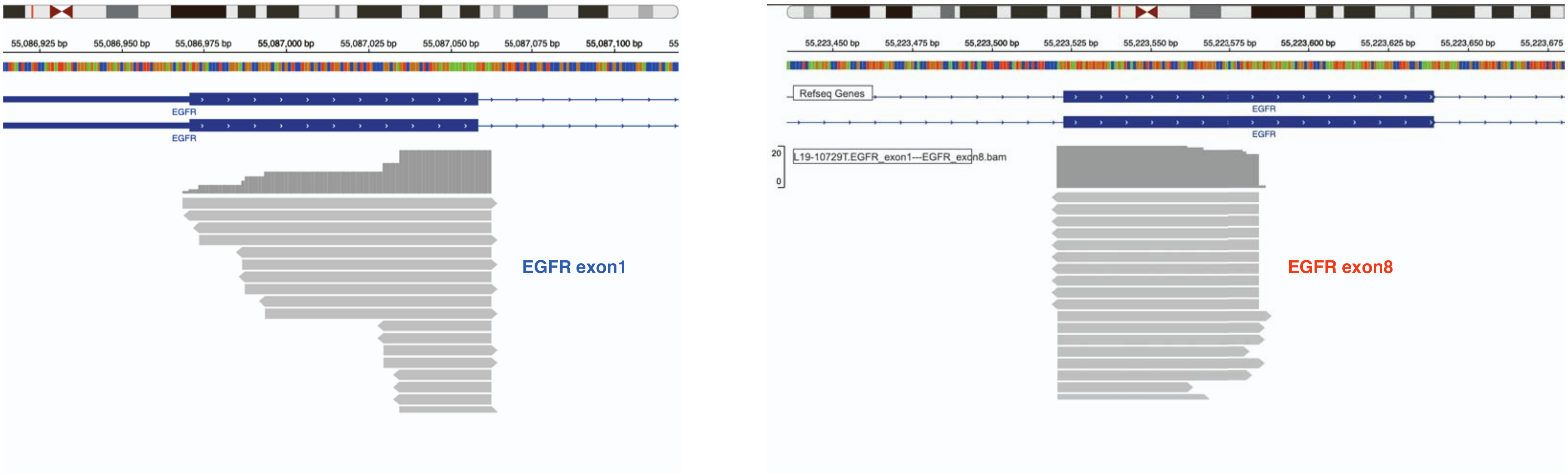

Figure S4.
Visualization of EGFR *vIII* showed the transcript with exons 2 - 7 deletion in a glioblastoma sample (targeted exon 8 colored in red and detected parter exon 1 in blue).

TCGA Case: PRAD-CH-5751

Variant 1: AR_exon3::AR_intronic, chrX_67686126__chrX_67694673

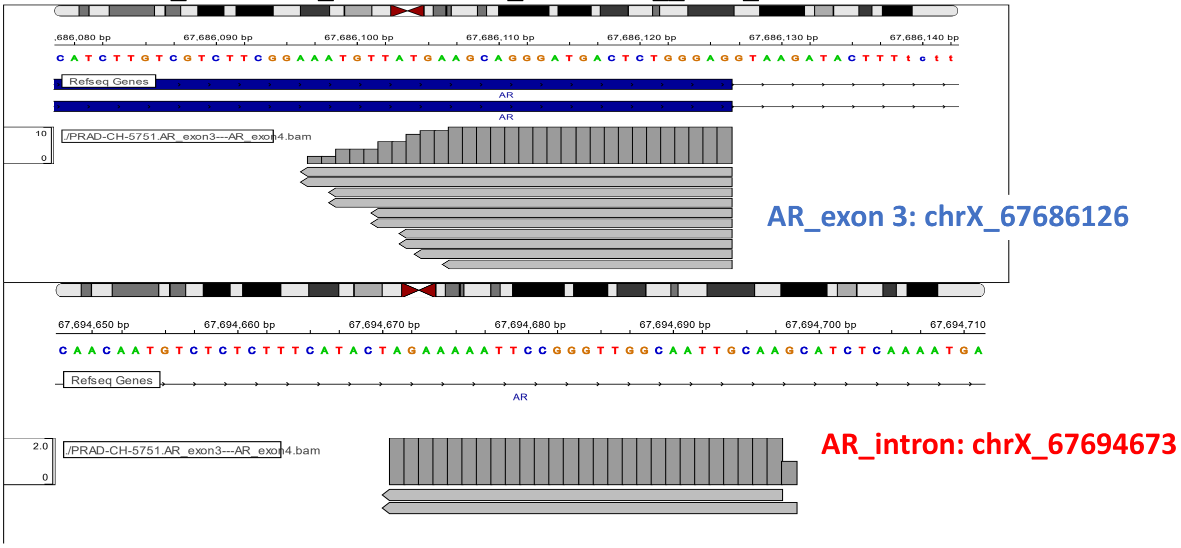

Variant 2: AR_exon3::AR_intronic, chrX_67686126__chrX_67689556.

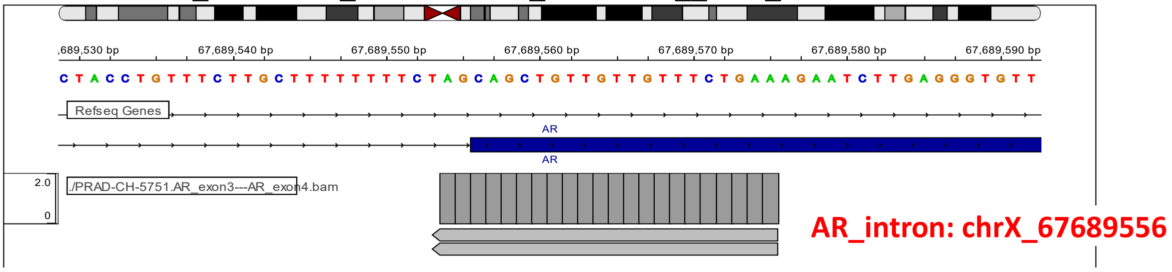

TCGA Case: PRAD-CH-5761

AR_exon3::AR_intronic, chrX_67686126__chrX_67694673

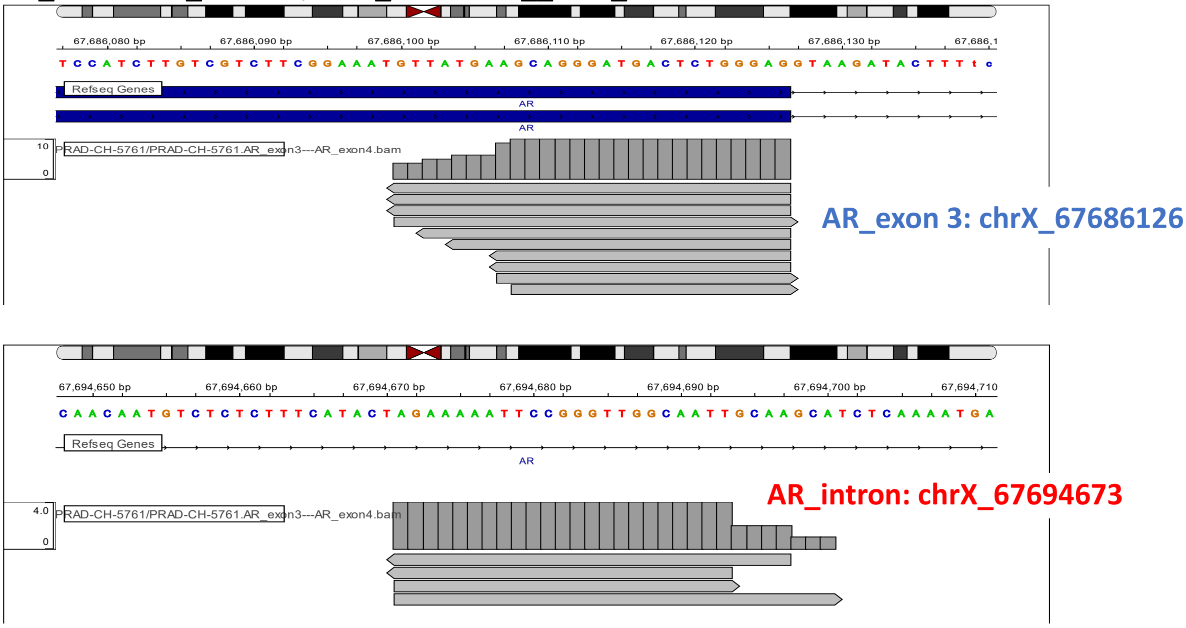

Figure S5. Two TCGA prostate adenocarcinoma (PRAD) cases with *ARv7* variants detected by SplitFusion.

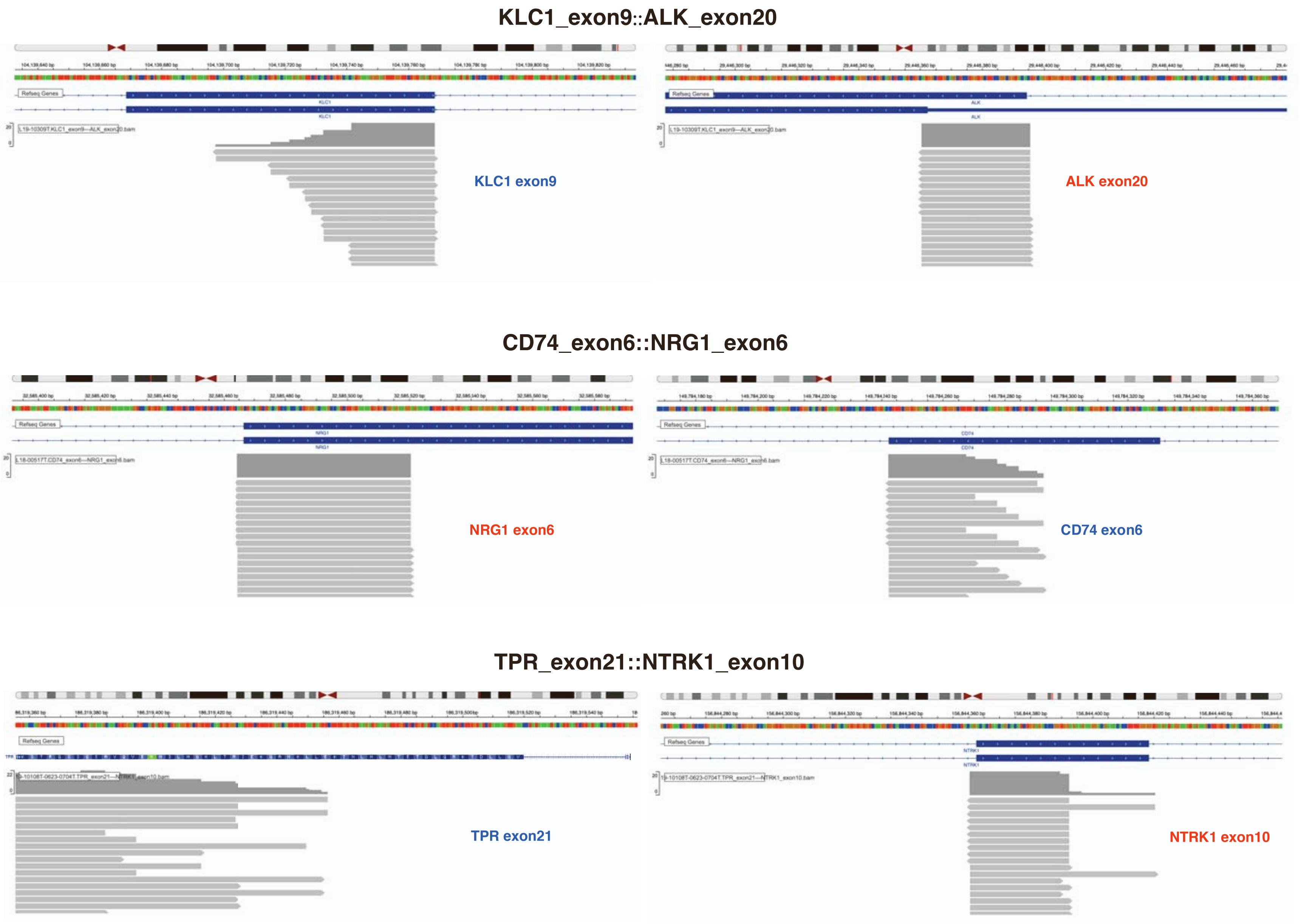

Figure S6. Visualization of rare fusions in lung cancer showed typical anchored multiplex PCR detected fusions involving targeted genes (colored in red; rectangle pileup) and detected fusion partners (colored in blue; open-end pileup) for *KLC1::ALK*, *CD74::NRG1*, and *TPR::NTRK1*. All of the fusion partners showed the alignments on exon boundary.

>FGFR3 exon 17::JAKMIP1 exon 4

GACCGTGTCCTTACCGTGACGTCCACCGACATGGATGAGATCAAAGGGAAAGACCGTGTGATTCTGGCCTTGGAGAAGGAACTTGGCGTGCAGGCTGGGCAGACC

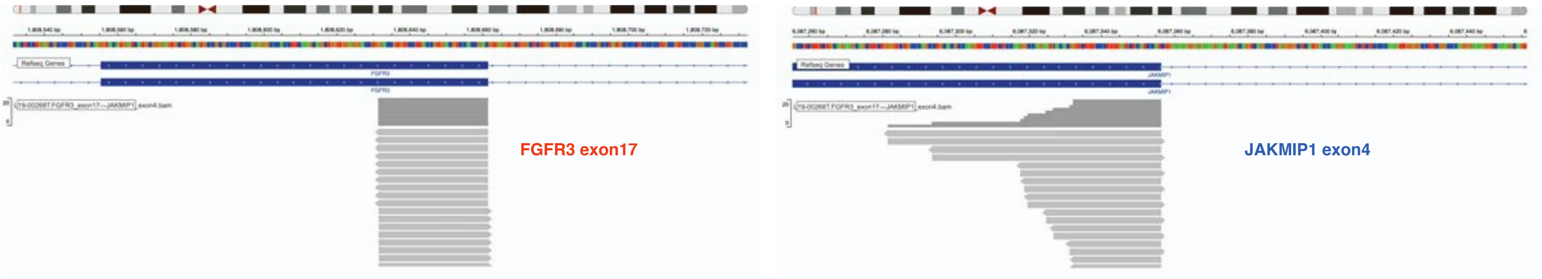

>CLIP2 exon 9::BRAF exon 9

TTGAGACCTTGAGACCCAGACGCAGCTGGAGCACGCGCGCATTGGGGAGCTGGAACAGAGCCTGCTACTGGAGAAGGCGCAGGCCGAGCGGCTGCTCCGAGAATTAGC G
GACAACAGGGACTTGATTAGAGACCAAGGATTTCGTGGTGA

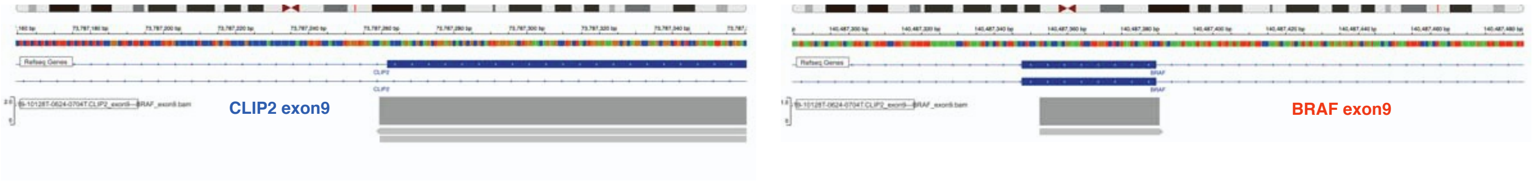

>CLIP2 exon 8::BRAF exon 9

CTGGAGGAGGAGAGGAGGAAGGTGGAGGATCTGCAGTTCCGCGTGGAGGAGGAGTCCATCACCAAGGGAGACCTGGAGGACTTGATTAGAGACCAAGGATTTCGTGGTGA

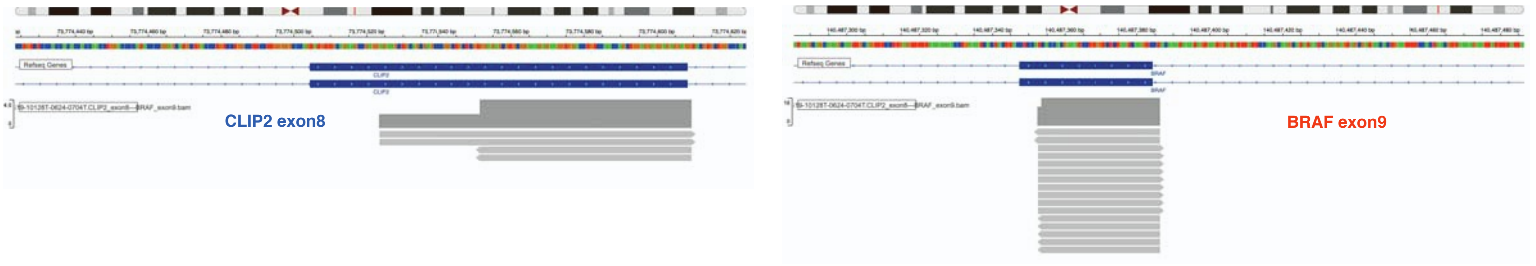

>ITPR2 exon 37::ETV6 exon 2

ACTGGGCTCTCTGGAGGTGTATATGAAATTCGTTCCTGCTCTTCCACAAAGCTGTCTTTCTTCTCTAACATTTCTCGTAATATCTGAAGAATTTTAATGCACAGTTTTTCTTC
TTTCTCCATTAGTTTCTTTGTATGATTAATCAACTTC

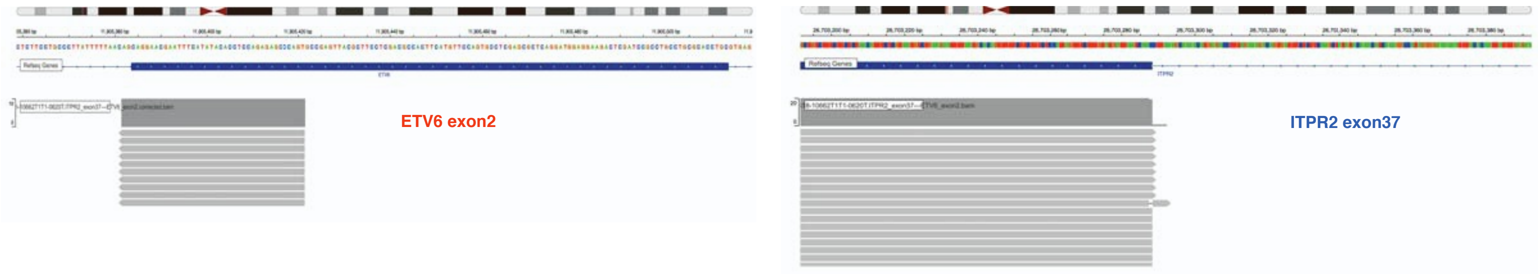

Figure S7. Visualization of new fusions in lung cancer showed typical anchored multiplex PCR detected fusions involving targeted genes (colored in red; rectangle pileup) and detected fusion partners (colored in blue; open-end pileup) for *FGFR3::JAKMIP1*, *CLIP2::BRAF*, and *ETV6::ITPR2*. All fusion partners showed the alignments on exon boundary.

MGH Case 1: CIC_exon20::DUX4_exon1

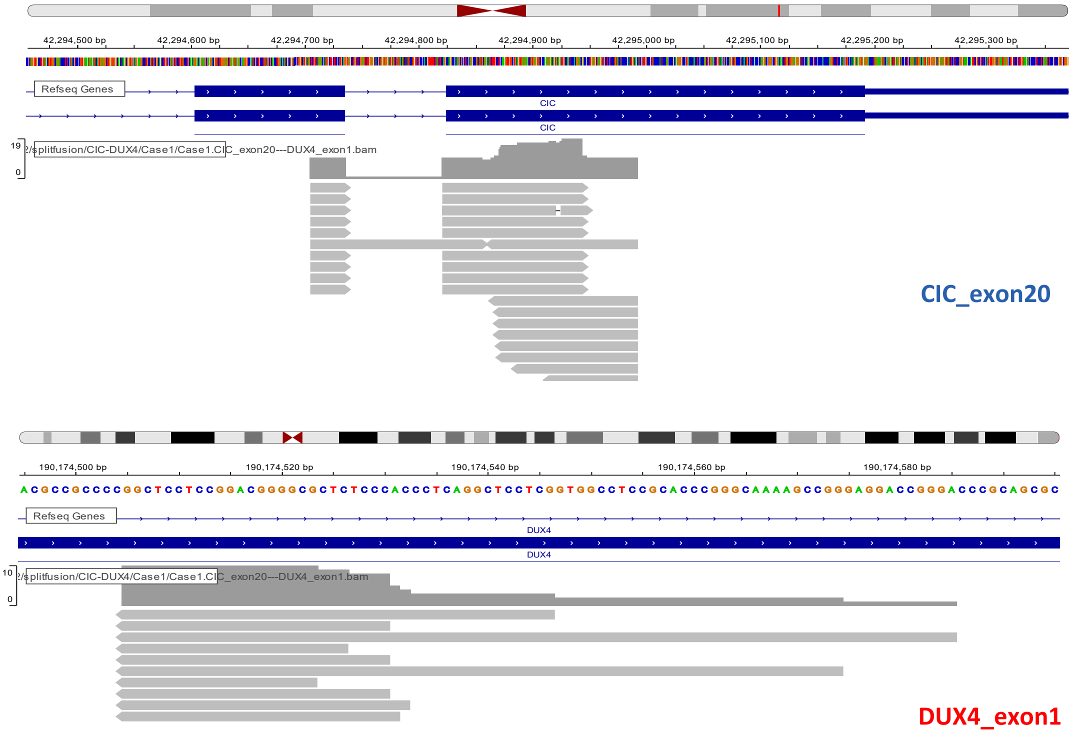

MGH Case 2: CIC_exon20::DUX4_exon1

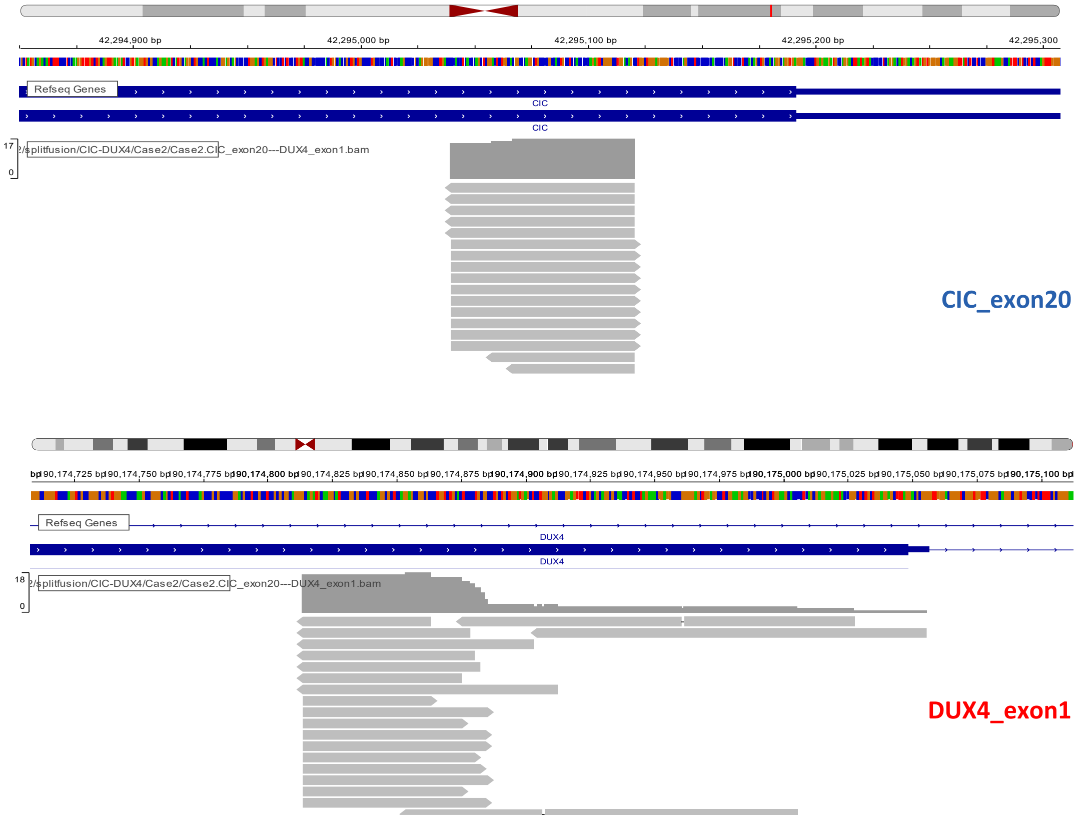

Figure S8. Visualization of two MGH clinical sarcoma cases with *CIC::DUX4* fusions detected by SplitFusion.

TCGA Case: LUAD-05-5428

Variant 1: SLC34A2_exon13::ROS1_exon32

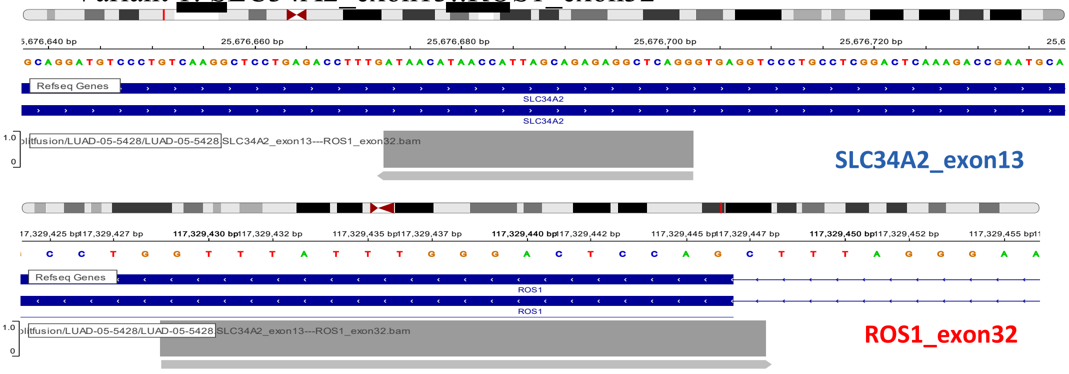

Variant 2: SLC34A2_exon13::ROS1_exon34

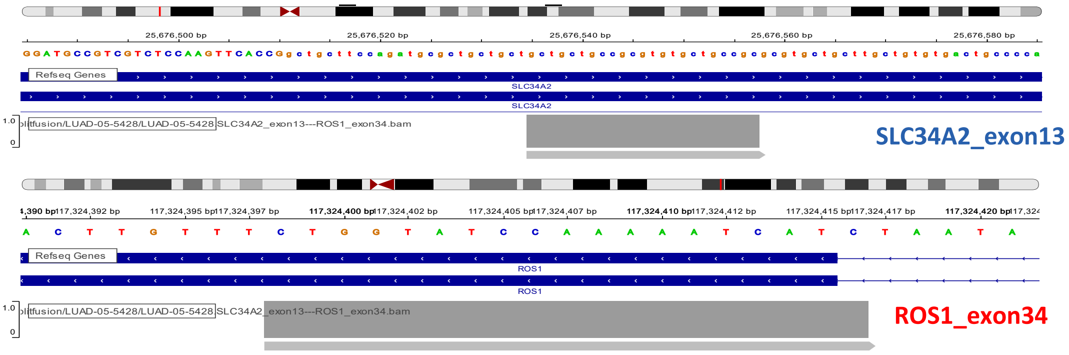

TCGA Case: LUAD-55-7816

Variant 1:SLC34A2_exon13::ROS1_exon32

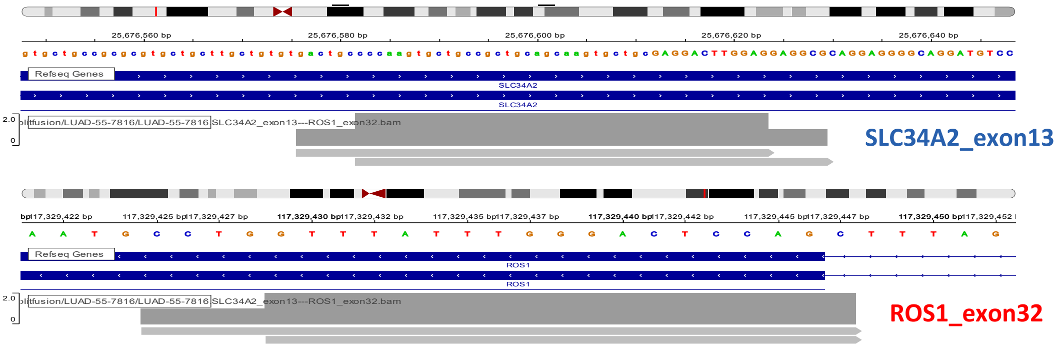

Variant 2: SLC34A2_exon13::ROS1_exon34

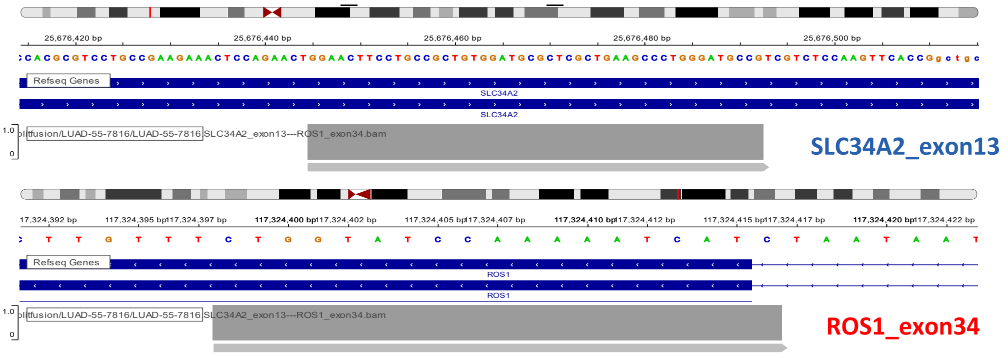

Figure S9. Visualization of two TCGA lung adenocarcinoma (LUAD) cases with *SLC34A2::ROS1* fusions detected by SplitFusion.

TCGA case:LUAD-69-7978

TUBD1_UTR5::RPS6KB1_exon2

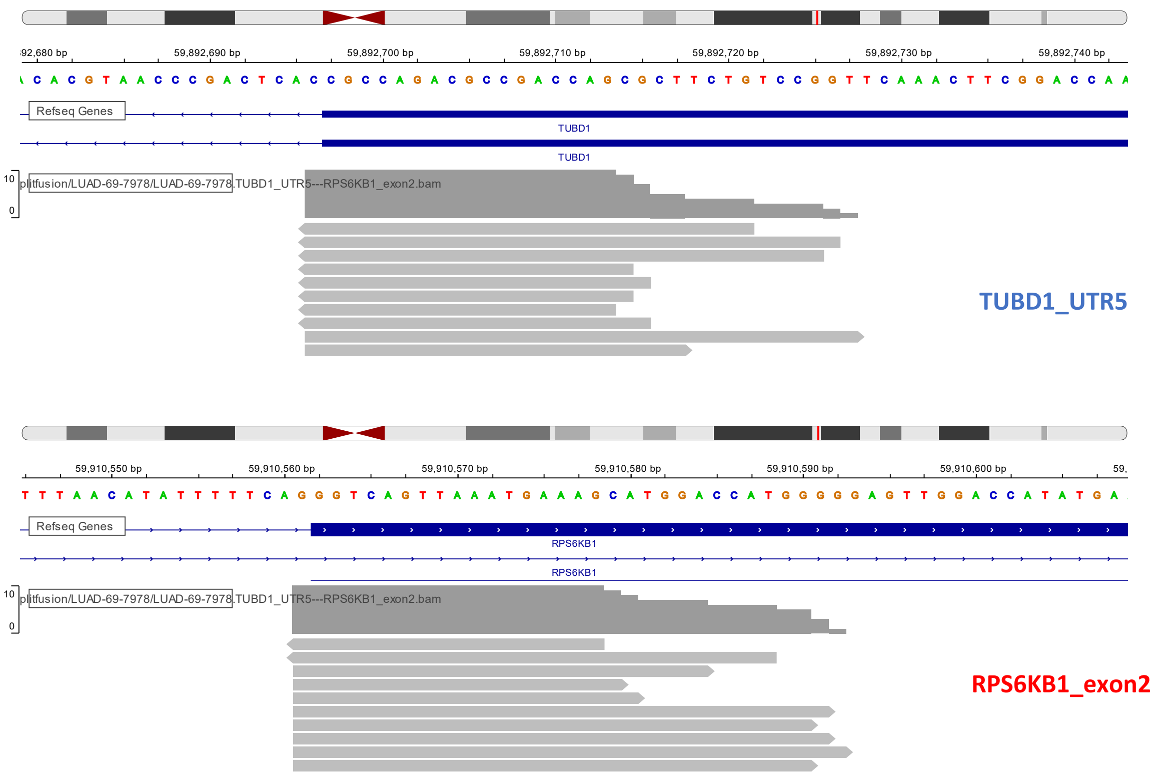

Figure S10. Visualization of a TCGA lung adenocarcinoma case with fusion involving 5'UTR of TUBD1 and exon 2 of RPS6KB1 detected by SplitFusion.

Table S1. The list of RNA and DNA targets in a 19-gene lung cancer panel.

RNA targets

Gene	NM	Exon	Sense /antisense
ALK	NM_004304	19,20,21,22	antisense
EGFR	NM_005228	1,8,9	antisense
FGR	NM_005248	1,2,3	antisense
MET	NM_000245	13,14,15	antisense
NRG1	NM_004495	1,2,3,4,6	antisense
NTRK1	NM_002529	8,10,11,12,13,14,15	antisense
NTRK2	NM_006180	11,12,13,14,15,16,17	antisense
NTRK3	NM_002530	13,14,15,16	antisense
ROS1	NM_002944	31,32,33,34,35,36,37	antisense
RET	NM_020630	8,9,10,11,12,1	antisense

DNA targets (Reference: hg19)

Gene	NM	start	end	chr
ALK	NM_004304	29432648	29432747	2
ALK	NM_004304	29443568	29443704	2
ALK	NM_004304	29445206	29445277	2
MSH2	NM_000251	47630328	47630544	2
MSH2	NM_000251	47635536	47635697	2
MSH2	NM_000251	47637229	47637514	2
MSH2	NM_000251	47639549	47639702	2
MSH2	NM_000251	47641404	47641560	2
MSH2	NM_000251	47643431	47643571	2
MSH2	NM_000251	47656877	47657083	2
MSH2	NM_000251	47672683	47672799	2
MSH2	NM_000251	47690166	47690296	2
MSH2	NM_000251	47693793	47693950	2
MSH2	NM_000251	47698100	47698204	2
MSH2	NM_000251	47702160	47702412	2
MSH2	NM_000251	47703502	47703713	2
MSH2	NM_000251	47705407	47705661	2
MSH2	NM_000251	47707831	47708013	2
MSH2	NM_000251	47709914	47710090	2
ROS1	NM_002944	117638302	117638438	6
ROS1	NM_002944	117639347	117639418	6
ROS1	NM_002944	117641027	117641196	6
EGFR	NM_005228	55241610	55241739	7
EGFR	NM_005228	55242411	55242516	7
EGFR	NM_005228	55248982	55249174	7
EGFR	NM_005228	55259408	55259570	7
MET	NM_000245	116411899	116412046	7
MET	NM_000245	116417439	116417526	7
MET	NM_000245	116423354	116423526	7
MET	NM_000245	116435937	116436180	7
BRAF	NM_004333	140453071	140453196	7
BRAF	NM_004333	140481372	140481496	7
FGFR1	NM_015850	38271432	38271544	8
FGFR1	NM_015850	38272073	38272150	8
FGFR1	NM_015850	38279311	38279462	8
FGFR1	NM_015850	38285866	38285956	8
CDKN2A	NM_000077	21968225	21968244	9
CDKN2A	NM_000077	21970897	21971210	9
CDKN2A	NM_000077	21974673	21974828	9
KRAS	NM_004985	25362726	25362848	12
KRAS	NM_004985	25378544	25378710	12
KRAS	NM_004985	25380164	25380349	12
KRAS	NM_004985	25398204	25398320	12
POLE	NM_006231	133250157	133250296	12
POLE	NM_006231	133253128	133253242	12
TP53	NM_000546	7572924	7573011	17
TP53	NM_000546	7573923	7574036	17
TP53	NM_000546	7576849	7576929	17
TP53	NM_000546	7577015	7577158	17
TP53	NM_000546	7577495	7577611	17
TP53	NM_000546	7578173	7578292	17
TP53	NM_000546	7578367	7578557	17
TP53	NM_000546	7579308	7579593	17
TP53	NM_000546	7579696	7579724	17
TP53	NM_000546	7579835	7579914	17
ERBB2	NM_004448	37871535	37871615	17
ERBB2	NM_004448	37880975	37881167	17
ERBB2	NM_004448	37881298	37881460	17
ERBB2	NM_004448	37882811	37882915	17
SMAD4	NM_005359	48573414	48573668	18
SMAD4	NM_005359	48575052	48575233	18
SMAD4	NM_005359	48575661	48575697	18
SMAD4	NM_005359	48581147	48581366	18
SMAD4	NM_005359	48584491	48584617	18
SMAD4	NM_005359	48584706	48584829	18
SMAD4	NM_005359	48586232	48586289	18
SMAD4	NM_005359	48591789	48591979	18
SMAD4	NM_005359	48593385	48593560	18
SMAD4	NM_005359	48603004	48603149	18
SMAD4	NM_005359	48604622	48604839	18

Table S2. The list of RNA and DNA targets in a 62-gene lung cancer panel.

RNA targets

Gene	Exon	Sense /antisense
ALK	18	antisense
ALK	19	antisense
ALK	20	antisense
ALK	21	antisense
B2M	2	antisense
B2M	2	sense
BRAF	8	antisense
BRAF	9	antisense
BRAF	10	antisense
BRAF	11	antisense
BRAF	12	antisense
CD274	2	antisense
CD274	3	antisense
CD274	4	antisense
CD274	4	sense
CD274	5	sense
CD274	6	antisense
CHMP2A	3	antisense
CHMP2A	3	sense
CSF1R	9	antisense
CSF1R	10	antisense
CSF1R	11	antisense
CSF1R	12	antisense
CSF1R	13	antisense
CSF1R	14	antisense
CTLA4	3	antisense
EGFR	1	antisense
EGFR	8	antisense
EGFR	9	antisense
EGFR	23	sense
EGFR	24	sense
EGFR	25	sense
ESR1	3	sense
ESR1	4	sense
ESR1	5	sense
ESR1	6	sense
ETV6	1	sense
ETV6	2	antisense
ETV6	2	sense
ETV6	3	antisense
ETV6	3	sense
ETV6	4	sense
ETV6	5	antisense
ETV6	5	sense
ETV6	6	antisense
ETV6	6	sense
ETV6	7	antisense
FGFR1	2	antisense
FGFR1	3	antisense
FGFR1	4	antisense
FGFR1	5	antisense
FGFR1	6	antisense
FGFR1	7	antisense
FGFR1	8	antisense
FGFR1	9	antisense
FGFR1	10	antisense
FGFR1	11	antisense
FGFR1	17	sense
FGFR2	2	antisense
FGFR2	8	antisense
FGFR2	9	antisense
FGFR2	10	antisense
FGFR2	17	sense
FGFR3	8	antisense
FGFR3	9	antisense
FGFR3	10	antisense
FGFR3	17	sense

Gene	Exon	Sense /antisense
FGFR4	2	antisense
FGFR4	8	antisense
FGFR4	9	antisense
FGFR4	10	antisense
FGFR4	17	sense
FGR	1	antisense
FGR	2	antisense
FGR	3	antisense
GZMA	2	antisense
GZMA	3	antisense
GZMA	4	antisense
GZMA	5	antisense
MET	13	antisense
MET	14	antisense
MET	15	antisense
NRG1	1	antisense
NRG1	2	antisense
NRG1	3	antisense
NRG1	4	antisense
NRG1	6	antisense
NTRK1	8	antisense
NTRK1	10	antisense
NTRK1	11	antisense
NTRK1	12	antisense
NTRK1	13	antisense
NTRK1	14	antisense
NTRK1	15	antisense
NTRK2	11	antisense
NTRK2	13	antisense
NTRK2	14	antisense
NTRK2	15	antisense
NTRK2	16	antisense
NTRK2	17	antisense
NTRK3	13	antisense
NTRK3	13	sense
NTRK3	14	antisense
NTRK3	14	sense
NTRK3	15	antisense
NTRK3	15	sense
NTRK3	16	antisense
PDCD1	2	antisense
PDCD1	3	antisense
PDGFRA	9	antisense
PDGFRA	10	antisense
PDGFRA	11	antisense
PDGFRA	12	antisense
PDGFRA	13	antisense
PDGFRA	14	antisense
PRF1	2	antisense
PRF1	3	antisense
RET	8	antisense
RET	9	antisense
RET	10	antisense
RET	11	antisense
RET	12	antisense
RET	13	antisense
ROS1	31	antisense
ROS1	32	antisense
ROS1	33	antisense
ROS1	34	antisense
ROS1	35	antisense
ROS1	36	antisense
ROS1	37	antisense
TRAC	1	antisense
TRBC	1	antisense

DNA targets

Selected hotspot exons

ABL1	FLT3	MPL
AKT1	FOXL2	NRAS
APC	GNA11	PIK3CA
ATM	GNAQ	POLE
AURKA	GNAS	PTEN
CDK4	H3F3A	PTPN11
CDKN2A	HRAS	RHOA
CTNNB1	IDH1	SMAD4
ERBB2	IDH2	TERT
ERBB3	JAK2	TP53
EZH2	KIT	
FBXW7	KRAS	
